# Saturating the Matrix: Nanocomposite Solution-Processed Sodium Aluminophosphate Solid Electrolytes

**DOI:** 10.1021/acsaem.5c02284

**Published:** 2025-10-01

**Authors:** Thomas E. Gill, Guillaume Matthews, Yaoguang Song, Mo El Maoued, Adam J. Lovett, Sadia Sheraz, Nicholas P. Lockyer, Amita Ummadisingu, Thomas S. Miller, Alexander J. E. Rettie

**Affiliations:** † Electrochemical Innovation Lab, Department of Chemical Engineering, 4919 University College London, London WC1E 6DH, U.K.; ‡ Advanced Propulsion Lab, University College London, Marshgate, Stratford E20 2AE, U.K.; § Department of Materials, 6396 University of Oxford, Oxford OX1 3PH, U.K.; ∥ Manufacturing Futures Lab, Department of Chemical Engineering, University College London, London WC1E 6DH, U.K.; ⊥ Department of Chemistry, Photon Science Institute, 5292 The University of Manchester, Oxford Road, Manchester M13 9PL, U.K.

**Keywords:** nanocomposite, amorphous, solid electrolyte, thin film, scalable

## Abstract

Amorphous lithium solid electrolytes (SE) have enabled high performance lithium metal batteries, but sodium analogues are underexplored. Here, we report sodium aluminophosphate (NAPO) SEs synthesized via spin coating from aqueous solutions. Continuous, smooth, films with submicron thickness are produced after a mild annealing step. Exploration of the Na–Al–P–O phase space reveals nanocomposite materials comprising of an amorphous NAPO with crystalline NaNO_3_ domains, suggesting a Na^+^ saturation limit within the Al–P–O matrix. A maximum ionic conductivity of ≈10^–8^ S cm^–1^ is achieved, with the presence of the insulating NaNO_3_ precursor necessary for high ionic conductivity. Electron microscopy, time-of-flight secondary ion mass spectrometry and optical measurements reveal that at low concentrations the NaNO_3_ phase is initially present as diffuse nanoparticle domains and at higher concentrations it forms isolated micron-sized particles. The optimal NAPO SE has an activation energy of 0.80(1) eV, a moderate reduced Young’s modulus ≈30 GPa and low electronic conductivity (≈10^–14^ S cm^–1^), making these materials promising candidates for artificial solid electrolyte interphases or as solid electrolytes in sodium metal batteries.

## Graphical abstract

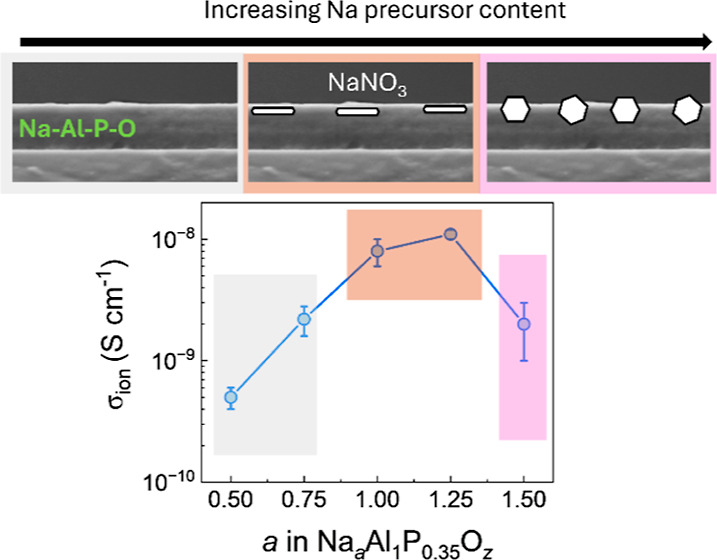

## 1 Introduction

Sodium-ion batteries (SIB) represent a promising alternative to lithium-ion batteries, due to the relative abundance and low-cost of sodium-based raw materials. However, the inherent reduction in energy density is a major drawback to the performance of SIBs. Integration of a Na metal anode (NMA) represents an avenue to maximize energy density, owing to its superior theoretic capacity versus commonly used hard carbon anodes (1166 vs 335 mAh g^–1^).^1^ However, the growth of metallic filaments (dendrites) during cycling poses a safety concern when integrating a NMA into cells with a flammable liquid electrolyte.

In light of this, there has been extensive research into crystalline sodium solid electrolytes (SEs) with ionic conductivities comparable to liquid counterparts.^2^ Despite their mechanical toughness, Na SEs are susceptible to penetration by Na metal filaments that lead to cell failure.^3^ There are notable exceptions to this, for example, Tsai et al.^4^ reported a Na_3.4_Zr_2_(SiO_4_)_2.4_(PO_4_)_0.6_ (NZSP) solid electrolyte that demonstrated excellent dendrite inhibition, without any postprocessing or applied pressure. In contrast, several other reports claim that untreated NZSP-type SEs suffer from dendrite induced failure at low currents,^5–7^ implying a complex relationship between SE properties and dendrite growth. Postprocessing of sodium-β″-alumina has also been reported to enable dendrite tolerance at high current densities.^8^ However, this was achieved with a high temperature (900 °C) treatment step in an inert atmosphere, which is undesirable for large scale production.

The amorphous thin-film SE, lithium phosphorus oxynitride (LiPON) has been reported to exhibit stable Li plating/stripping for 10,000 cycles,^9^ as well as high-rate capabilities (>10 mA cm^–2^) when utilized in thin-film microbatteries.^10^ This excellent tolerance to dendrite growth has been linked to key properties such as a low electronic conductivity,^11^ as well as the absence of grain boundaries in the amorphous thin film.^12^ LiPON has also been reported to produce a stable and low impedance interphase when in contact with Li metal.^13^ Owing to these properties, LiPON has been studied as a stabilizing interfacial layer or artificial solid-electrolyte-interphase—increasing the dendrite tolerance of fast ion conducting SEs,^14,15^ as well as improving the cyclability of Li metal full cells with liquid electrolytes.^16,17^

These reports prompted the development of sodium phosphorus oxynitride (NaPON), which was recently shown to be electrochemically stable against a NMA.^18–20^ The major drawback of both LiPON and NaPON is the reliance on slow and expensive vacuum deposition techniques, which restricts their scalability and applicability to large-format cells. The development of amorphous Na thin-film SEs synthesized via scalable processing is therefore of significant interest. Previous work^21,22^ has demonstrated that spin-coating inorganic nanoclusters from aqueous solutions with a mild annealing step (<500 °C) can yield smooth, dense, homogeneous Li-based films with desirable SE properties. Inspired by these studies, we report the synthesis of Na–Al–P–O (NAPO) thin film SEs. Avoiding the use of organic solvents is desirable to reduce costs and improve the sustainability of the manufacturing process.

First, we synthesize new NAPO thin film SEs and measure their physical, mechanical, structural and ionic conduction properties. Second, we explore the Na–Al–P–O phase space, revealing the presence of a two-phase system, comprising NaNO_3_ crystallites embedded in an amorphous NAPO matrix. A maximum room temperature ionic conductivity of ≈10^–8^ S cm^–1^ is determined for some of these nanocomposites. The optimal composition has desirable mechanical and electronic properties for use as a thin solid electrolyte coating in advanced batteries.

## 2 Experimental Methods

### 2.1 Material Preparation

NAPO thin films were prepared via spin coating from aqueous solutions. In a typical synthesis, precursor solutions were prepared by completely dissolving 25 mmol of Al­(OH)_3_·*x*H_2_O (>99%, Thermo Fisher Scientific) in 20 mL of deionized (DI, 18 MΩ) water. The level of hydration was determined to be *x* = 0.9 via thermogravimetric analysis. To these solutions, 37.5 mmol of HNO_3_ (70% aq, >99.9%, Sigma-Aldrich) and 12.5 mmol of H_3_PO_4_ (85% aq, >99.9%, Sigma-Aldrich) were added and stirred overnight at 80 °C to allow complete dissolution. After cooling to room temperature, 25 mmol of NaNO_3_ (>99%, Fisher Scientific) was added and stirred at 50 °C for 24 h. The NaNO_3_ and H_3_PO_4_ amounts were adjusted in order to obtain a range of compositions with various Na/Al/P molar ratios. Finally, all solutions were diluted with DI water to achieve a final concentration of 0.8 M with respect to Al.

Silicon (Si) wafers (p-type, ⟨100⟩, boron-doped, single-side polished, resistivity <0.01 Ω cm, PI-KEM) were used as a substrate and bottom electrode. The Si substrates were cut into 2 × 2 cm^2^ samples with a diamond scribe, and ultrasonicated in a series of acetone, IPA and DI water, before being dried under nitrogen. To remove any remaining surface contaminants the substrates were O_2_ plasma-treated (Henniker HPT-100) at 100 W for 5 min, resulting in a clean hydrophilic surface.

The precursor solution was then sonicated at 50 °C for 30 min. Once cooled to room temperature, the substrate was flooded with solution through a 0.2 μm Teflon filter attached to a syringe, spin coated at 3000 rpm for 30 s and immediately transferred to a preheated hot plate at 275 °C for 5 min. After removing the samples and allowing them to cool to room temperature, the process was repeated to obtain multilayer films. Unless stated otherwise, 2 layers were deposited for characterization studies. Once the final layer was deposited, a final 1 h anneal was conducted either at 275 °C using a hot plate or at 350–800 °C in a box furnace. All films were stored in an Ar-filled glovebox (H_2_O and O_2_ level <0.5 ppm) to minimize any contamination with atmospheric moisture.

A NaNO_3_ pellet (12 mm diameter, 5 mm thick) was prepared by placing vacuum-dried NaNO_3_ powder into a cylindrical pressing die and pressed with a uniaxial pressure ≈100 MPa at room temperature. The pellet was then calcinated at 275 °C for 1 h in air in a box furnace before being vacuum-dried overnight at 100 °C and stored in an Ar-filled glovebox.

### 2.2 Physicochemical Characterization

Film thickness was determined using a SEMILAB SE-2000 rotating compensator spectroscopic ellipsometer. Films deposited on Si substrates were measured under ambient conditions with an incident angle of 70° between 250 and 1700 nm wavelengths. The measured ellipsometry spectra (Figure S1 in the Supporting Information) were modeled and analyzed with the Cauchy dispersion law using Semilab SEA software to obtain the film thickness. Cross-sectional imaging was carried out using scanning electron microscopy (SEM, ZEISS, GeminiSEM 360). Samples for SEM were prepared by sputtering a thin layer of Au onto the surface, before being gently broken into <0.5 cm^2^ pieces. The surface morphology and mechanical properties were characterized using atomic force microscopy (AFM). AFM experiments were carried out using a Bruker Dimensions Icon with ScanAsyst housed in an Ar-filled glovebox (<0.5 ppm of H_2_O and O_2_) with Bruker RTESPA-150 (Sb (n) doped Si with reflective Al coating, *k* = 5 N m^–1^, *f* ^0^ = 150 kHz) or Bruker RTESPA-300 (Sb (n) doped Si with reflective Al coating, *k* = 40 N m^–1^, *f* ^0^ = 200 kHz) AFM tips. Two modes were used. Bruker’s ScanAsyst mode (peak force tapping) was used to characterize the film topography. The reduced Young’s modulus was mapped using the Quantitate NanoMechanics (QNM) PeakForce tapping mode using the relative mode calibrated against highly ordered pyrolytic graphite (18 GPa).^23,24^ Further information about the method can be found elsewhere.^25^ All AFM images were analyzed using the Gwyddion software.^26^

Scanning-transmission electron microscopy (STEM) analysis was performed by using a Zeiss Merlin instrument equipped with a field emission gun (FEG) operated at 30 kV. Prior to analysis, a suspension was prepared by adding a few mg of NAPO powder into 15 mL of acetone in a beaker which was subsequently ultrasonicated for 2 min. A lacey carbon copper grid was dipped into the suspension and dried to make the STEM sample. NAPO powder was obtained by scratching a single-layer NAPO film deposited onto a glass microscope slide. STEM micrographs were acquired with bright field imaging mode, and a low acceleration voltage was required to avoid beam damage of the specimen.

Grazing incidence X-ray diffraction (GI-XRD) was conducted with a Malvern PANalytical Empyrean diffractometer, using a Cu source. Films were deposited onto fused silica substrates, which were cleaned in the same way as described for Si wafers in Section 2.1. The fused silica substrates (1 mm thick) were purchased precut to 2 × 2 cm^2^ from Multilab. Data was collected with a fixed angle of incidence (1°) over a 2θ range of 10 to 50°.

Surface composition was determined using X-ray photoelectron spectroscopy (Thermo Fisher, Kα photoelectron spectrometer, Al source) with binding energies referenced against the adventitious carbon (C) 1s peak at 284.8 eV. The process was carried out under ultrahigh vacuum conditions (10^–7^ to 10^–6^ Pa). Survey scans were collected first before additional scans centered around regions containing elements of interest were conducted. In order to collect data with adequate signal-to-noise ratio, a minimum of 50 scans were used in the regions of Na, Al, P, O and N. CasaXPS software was used to analyze the data, and Shirley background fitting was used. An estimate of the surface-level elemental composition was determined from the peak areas, which were first normalized using the relative sensitivity factors from CasaXPS, Na (8.52), Al 2p (0.537), P 2p (1.192) and O 1s (2.93), and N 1s (1.8). Time of flight secondary ion mass spectrometry (ToF-SIMS) was conducted with an Ionoptika J105-SIMS instrument,^27^ using a 40 keV C_60_^+^ primary ion beam in positive and negative ion imaging mode. Samples were first heated to 50 °C inside the ToF-SIMS instrument prior to analysis. Depth profile analysis was then completed using a primary ion dose of 1.11 × 10^13^ ions cm^–2^ per layer with 128 × 128 pixels over an area of 150 μm × 150 μm until a total dose of 1.5 × 10^15^ ions cm^–2^ was reached. Diffuse reflectance measurements were obtained using a Shimadzu UV-2600i spectrophotometer with an integrating sphere accessory, in a wavelength range of 220–1400 nm with a 1 nm step-size.

### 2.3 Electronic and Electrochemical Characterization

Through-plane ionic conductivity measurements were performed throughout. Circular Al top contacts (1.13 mm^2^, ≈100 nm) were deposited by sputtering through a shadow mask, and Al foil was adhered to the back of the Si wafers using conductive Ag epoxy (Agar Scientific). An in-house cell holder was used to take conductivity measurements, where an Au-plated screw with a rounded tip gently contacted the Al contacts. Since thinner films could be susceptible to piercing under contact pressure from the screw, 2-layer films were used. The Au screw and Al foil were connected to a potentiostat (Reference 600+, Gamry) for electrochemical measurements. For studying the ionic conductivity of the NaNO_3_ pellet, a separate in-house cell was used, which consisted of two stainless steel pins connected in a PTFE holder which could be hand-pressed and screwed together to ensure good contact.^28^ Electrochemical impedance spectroscopy (EIS) was conducted using a 20 mV perturbation voltage over a frequency range of 100 mHz to 1 MHz for NAPO films, with a lower limit of 0.1 mHz used for the NaNO_3_ pellet. The EIS data were fit using an equivalent circuit model (ECM) consisting of a combination of electrical components in a Randles-type circuit.^29,30^ A resistor (*R*) was used to model the impedance originating from electrical contacts (*R*_o_), a constant phase element (CPE) accounted for electrode polarization due to nonsymmetric blocking electrodes (CPE_pol_). A “*RCPE*” unit consisting of a *R* and CPE in parallel was used to represent the relaxation process associated with the migration of mobile ions through the bulk of the solid electrolyte. From this, a value for the bulk resistance (*R*_b_) of the solid electrolyte could be estimated and the ionic conductivity, σ_ion_ calculated using eq 1

1 $$\sigma_{ion}=\frac{l}{R_{b}A}$$

where, *l* is the thickness of the film (as determined by ellipsometry), *R*_b_ is the bulk solid electrolyte resistance and *A* is the geometric area of the Al contact. An average was taken from 3 different films, with each film being sampled in multiple positions (>3) across the film. For the NaNO_3_ pellet, *l* was determined with a digital Vernier caliper and *A* was defined by the area of the pellet. Room temperature measurements were conducted inside an Ar-filled glovebox. All EIS measurements were conducted within 24 h of film synthesis. However, repeat tests after 1 month of storage in an Ar-filled glovebox indicated no significant change to the resistance. Temperature-dependent EIS measurements were carried out inside a thermal chamber in air, with data collected at defined temperatures after a 30 min wait-time to ensure thermal equilibrium. By fitting the data to an Arrhenius relationship (eq 2), the activation energy (*E*_a_) of the ion hopping process was determined

2 $$\sigma_{ion}T=\sigma_{o}\;e^{-E_{a}/kT}$$

where, σ_0_ is a pre-exponential factor, *E*_a_ is the activation energy, *k* is the Boltzmann constant (8.62 × 10^–5^ eV K^–1^), and *T* is temperature. For the DC polarization experiments, a voltage bias of 1 V was applied for 1 h and the current–voltage curve fit to an exponential decay function. A longer duration constant-voltage experiment was run over 12 h, which confirmed that 1 h was sufficient to reach steady state.

## 3 Results and Discussion

### 3.1 Synthesis

In a previous study,^22^ we reported a range of Li_*a*_Al_1_P_*c*_O_*z*_ thin films, with the nominal Li and P content in the precursor solution varied between 2.25 < *a* < 3.25 and 1.15 < *c* < 1.5. Attempts to incorporate Na^+^ into similar Al–P–O frameworks by direct substitution of NaNO_3_ for LiNO_3_ in these precursor solutions resulted in films of poor quality by visual inspection. Modifying both the Na and P contents to molar ratios of Na/Al/P = 1:1:0.5 yielded visibly homogeneous thin films, indicating different interactions between Na and the Al–P–O networks compared to Li. Annealing similarly deposited thin films at 275 °C for 1 h has been shown to be sufficient to dehydrate, condense and decompose Li–Al–P–O and Al–P–O phases deposited via spin coating from aqueous solutions.^21,22,31^ This is lower than the known decomposition temperature of pure lithium nitrate (640 °C),^32^ demonstrating lower thermal requirements for thin films compared to bulk powders. Therefore, despite the relatively higher decomposition temperature of pure NaNO_3_ (740 °C),^32^ a 275 °C annealing temperature was used to produce Na–Al–P–O (NAPO) thin films as well.

### 3.2 Physicochemical & Electrochemical Properties

First, the cross-sectional morphology of the deposited films was observed by SEM. From Figure 1a, the 7-layer film appears uniform and dense, with no clear signs of cracks or pinholes. The thickness determined from the SEM image was ≈500 nm for the 7-layer film, which was in good agreement with the thickness determined via ellipsometry (505 nm). Ellipsometry studies on 2-layer films revealed a thickness of 160 nm, indicating a thickness of ≈80 nm per layer. The surface topography of the deposited films was then studied with AFM (Figure 1b). The films were flat and continuous, with a low average surface roughness ≤1 nm. The reduced Young’s modulus was determined from quantitative nanomechanical AFM to be 28.1 GPa. This value is lower than LiPON (77 GPa),^33^ LAPO (42 GPa)^22^ and garnet oxides (140–160 GPa),^34^ and above typical sulfides (15–20 GPa).^35,36^ The value for NAPO calculated here is promising, as an intermediate Young’s modulus is considered important for the dendrite tolerance of LiPON.^33^

**Figure 1:**
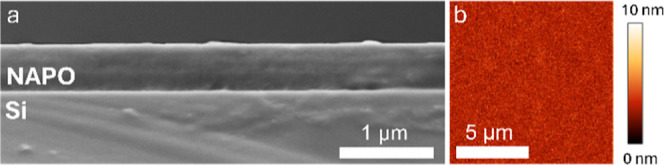
(a) Cross-sectional SEM image of a 7-layer Na_1_Al_1_P_0.5_O_*z*_ film on a Si substrate annealed at 275 °C. (b) AFM surface topography map of a 2-layer Na_1_Al_1_P_0.5_O_*z*_ film.

The surface chemical composition was then studied using X-ray photoelectron spectroscopy (XPS). The Na 1s, O 1s, Al 2p and P 2p spectra (Figure 2) for a film annealed at 275 °C showed the elements in their expected charge states of Na^+^, O^2–^, Al^3+^, and P^5+^. The N 1s spectra displayed a peak at around 407.5 eV, which is typical for N in a NaNO_3_ environment, indicating there was a fraction of the precursor salt that remained undecomposed after the 275 °C annealing process. This was examined further in the Na 1s spectra which could be fit with two peaks: the largest peak at 1071.9 eV was assigned to the Na^+^ in the NAPO matrix, with a secondary peak at 1070.4 eV assigned to Na^+^ in a NaNO_3_ environment. A normalized area ratio of ≈1:1 was determined for the NaNO_3_ peaks in the N 1s and Na 1s spectra, consistent with the elemental ratio N in NaNO_3_. Comparison of the peak areas between the two Na features indicated ≈10% of Na existed as NaNO_3_. The O 1s spectra contained a dominant peak at 531.4 eV which is characteristic of O within an AlPO_4_-type environment, with the higher energy peak at 536.6 eV associated with an Na Auger emission. There were also no clear indications of peaks associated with H_2_O (≈533 eV) or –OH (≈532 eV) groups, suggesting that NAPO is largely dehydrated after annealing, consistent with the previous reports on LAPO thin films synthesized via spin coating from aqueous solutions.^21,22^ The Al 2p and P 2p peaks at 74.5 and 133.8 eV respectively are consistent with an AlPO_4_-type environment.

**Figure 2:**
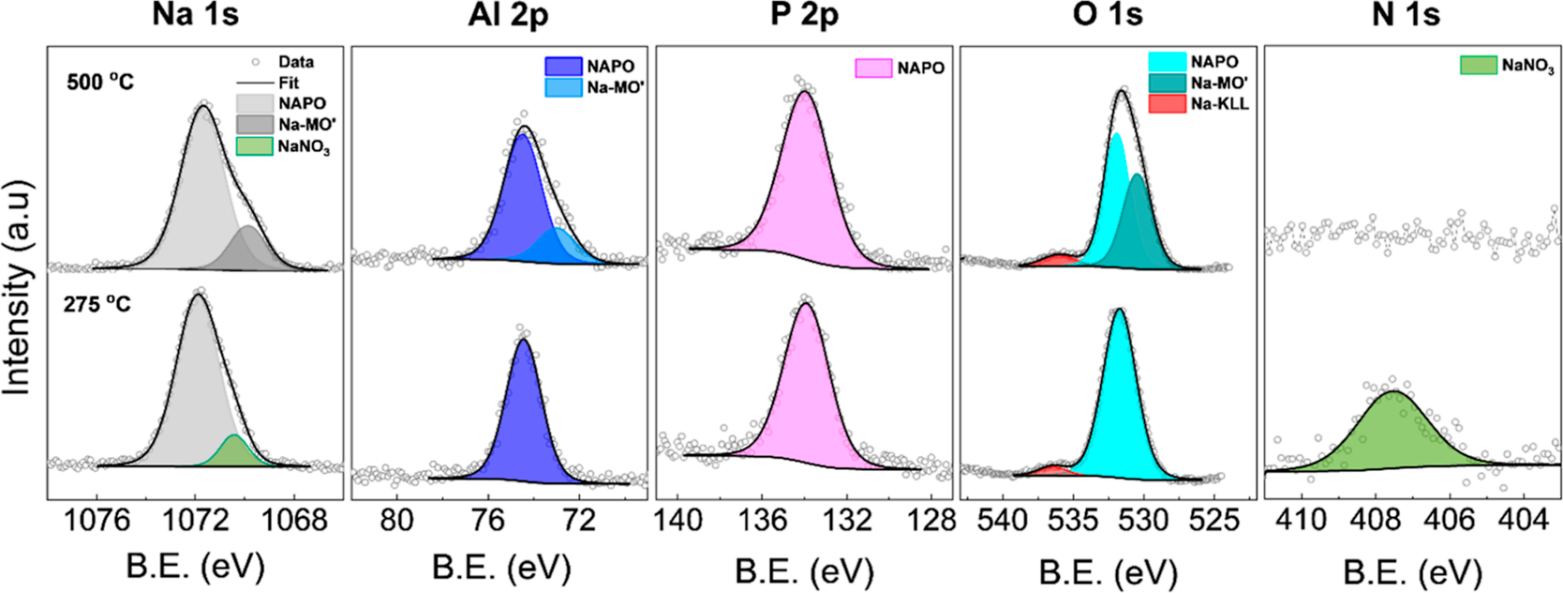
XPS data for Na_1_Al_1_P_0.5_O_*z*_ films after a 1 h anneal at 275 and 500 °C.

Preliminary experiments showed that the residual nitrate on the surface could be removed by increasing the final annealing temperature to 500 °C, as shown by the absence of the nitrate peak in the N 1s spectra. However, a different lower energy Na peak was observed in the Na 1s spectra, indicating the formation of a new secondary Na environment. The emergence of a secondary peak in the O 1s indicated the presence of separate oxide framework (Na-MO′), and a new shoulder peak in the Al 2p spectra indicated that a fraction of the Al^+^ is also integrated into this Na-MO′ structure. There were no new environments observed in the P 2p spectra, with the minor shifts in binding energy related to the change in the neighboring chemical environment after the removal of the residual nitrate. It is worth noting that an immediate 5 min cure at 275 °C was used prior to the final 1 h anneal at 500 °C. Therefore, this separate phase is likely a result of a secondary reaction that occurs during the 500 °C anneal, where the residual nitrate present after the preset anneal at 275 °C is decomposed into a new environment within the film. The surface elemental ratios determined via XPS (Table S1, Supporting Information) showed that the elemental Na and P content was consistent between different annealing temperatures.

The structural properties of films annealed at 275 and 500 °C were assessed using GI-XRD (Figure 3a). The data collected for both films showed a predominantly amorphous structure, which is consistent with the reports on related Li-containing materials.^21,22^ However, for the film annealed at 275 °C there was a weak peak around 29° which was indexed to crystalline NaNO_3_ powder (data experimentally collected). The lack of this feature at 500 °C is consistent with the loss of the nitrate peak in the N 1s spectrum.

**Figure 3:**
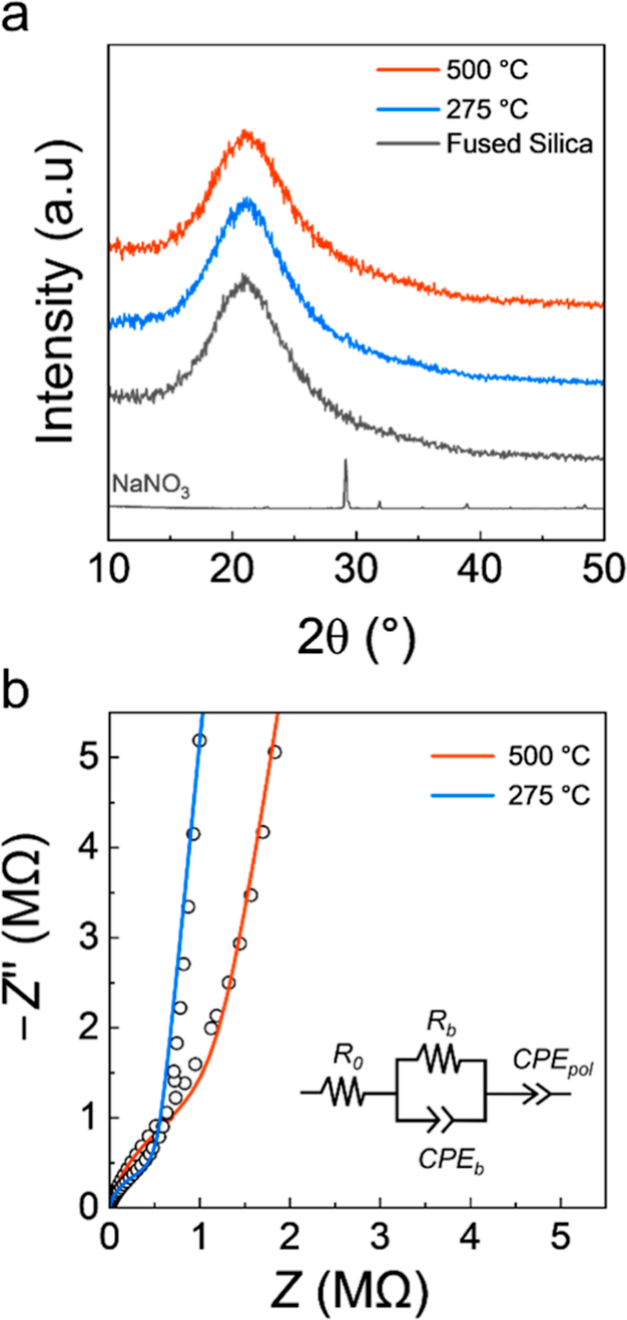
(a) GI-XRD data collected for NAPO films annealed at 275 and 500 °C, and NaNO_3_ precursor. (b) EIS data for NAPO films annealed 275 and 500 °C with equivalent circuit model used to fit the data.

The ionic conductivity (σ_ion_) of NAPO films annealed at 275 °C and 500 °C was determined using EIS. Although XPS showed no strong peaks associated with H_2_O or –OH, measures were taken to minimize any potential impact of water contamination due to hygroscopic nature of NaNO_3_. All films were immediately transferred from hot plate to a vacuum chamber to apply the Al electrodes. Samples were then heated at 150 °C for 1 h during the drying of Al back contacts before immediate transfer inside an Ar-filled glovebox for testing.

EIS data (Figure 3b) collected for both samples were adequately fit by the ECM shown inset in Figure 3b. A singular “*RCPE*” unit was used to model the conduction of Na^+^. This choice was supported by the presence of a singular peak in the Bode plots for both films (Figure S2 in the Supporting Information), indicating the presence of a single charge transfer process. As the annealing temperature increased from 275 to 500 °C, there was a small reduction in the ionic conductivity from 2.3(3) × 10^–9^ to 1.3(2) × 10^–9^ S cm^–1^. This agrees with previous work on a LAPO material, in which an inverse relationship between σ_ion_ and annealing temperature was observed.^22^

To understand the potential impact of the residual NaNO_3_ on the ionic conductivity, EIS data was also collected for a vacuum-dried NaNO_3_ pellet (Figure S3 in the Supporting Information). It should be noted that an elevated temperature of 40 °C was required to obtain meaningful data in this case. The bulk σ_ion_ was ≈10^–11^ S cm^–1^ at 40 °C, 2 orders of magnitude slower than the value reported for the two-phase Na_1_Al_1_P_0.5_O_*z*_ at room temperature. The higher σ_ion_ in the two-phase system despite the presence of insulating crystalline NaNO_3_ suggests that such particles may be beneficial to the conduction properties of the film. However, it is also possible the NaNO_3_ crystallites are isolated within the NAPO matrix and are not involved in the charge transport. Furthermore, the reduction in the σ_ion_ despite its removal at 500 °C may also be connected to structural changes that occur at the higher annealing temperature, causing less favorable pathways for ionic conduction.

### 3.3 Compositional Exploration

Variation of elemental composition has been shown to impact both ion transport and structural properties of bulk sodium aluminophosphate glasses.^37,38^ Therefore, a systematic exploration of the NAPO phase space was conducted in order to further study the two-phase systems observed in Section 3.2. This was achieved by adjusting the Na and P ratios relative to Al in Na_*a*_Al_1_P_*c*_O_*z*_ while keeping the annealing temperature constant at 275 °C. Note that *a* and *c* represent the nominal Na and P content in the precursor solutions. First, fixing the Na content at *a* = 1, the P content in Na_1_Al_1_P_*c*_O_*z*_ films was varied between 0.2 < *c* < 1.0. The analysis of the collected N 1s XPS spectra revealed a complete loss of the nitrate peak as P content was increased to *c* = 1.0. The GI-XRD data (Figure 4b) displays a predominantly amorphous framework for all NAPO phases, with the presence of crystalline NaNO_3_ evident for phases *c* ≤ 0.5. The increased peak intensity for *c* ≤ 0.35 suggests a greater presence of NaNO_3_ crystallites within these NAPO matrices. From the XPS and XRD data for *c* ≥ 0.8 it is apparent that a 275 °C anneal was sufficient to achieve extensive, if not complete, decomposition of NaNO_3_. Therefore, it can be inferred that for *c* < 0.8 the Al–P_*c*_–O_*z*_ matrices are likely saturated with Na^+^ ions, and the excess crystallized out in the form of NaNO_3_. From the fitted EIS data (Figure S4, Supporting Information) the σ_ion_ (Figure 5) was shown to increase from 1.5(6) × 10^–10^ S cm^–1^ to 8(2) × 10^–9^ S cm^–1^ as the P content was reduced from *c* = 1.0 to 0.35, before decreasing to 4(1) × 10^–9^ S cm^–1^ at *c* = 0.2. A maximum σ_ion_ was achieved for a composition of Na_1_Al_1_P_0.35_O_*z*_, which consisted of an amorphous NAPO phase and crystalline NaNO_3_ phase. Since this σ_ion_ value is ∼3 orders of magnitude higher than the calculated bulk σ_ion_ for NaNO_3_, we assume that the NaNO_3_ crystallites are likely isolated within the more conductive amorphous NAPO matrix. This indicates that for improved ionic conductivity it was desirable to saturate the NAPO matrix to the point that NaNO_3_ crystallites appear in the materials.

**Figure 4:**
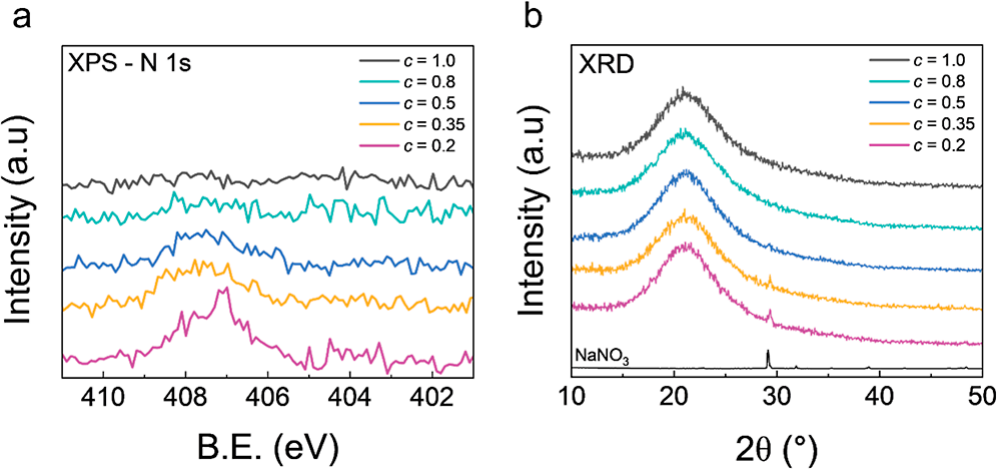
(a) N1 XPS spectra, (b) GI-XRD data for Na_1_Al_1_P_*c*_O_*z*_ films with varied P content from 0.2 < *c* < 1.0 and a fixed annealing temperature of 275 °C.

**Figure 5:**
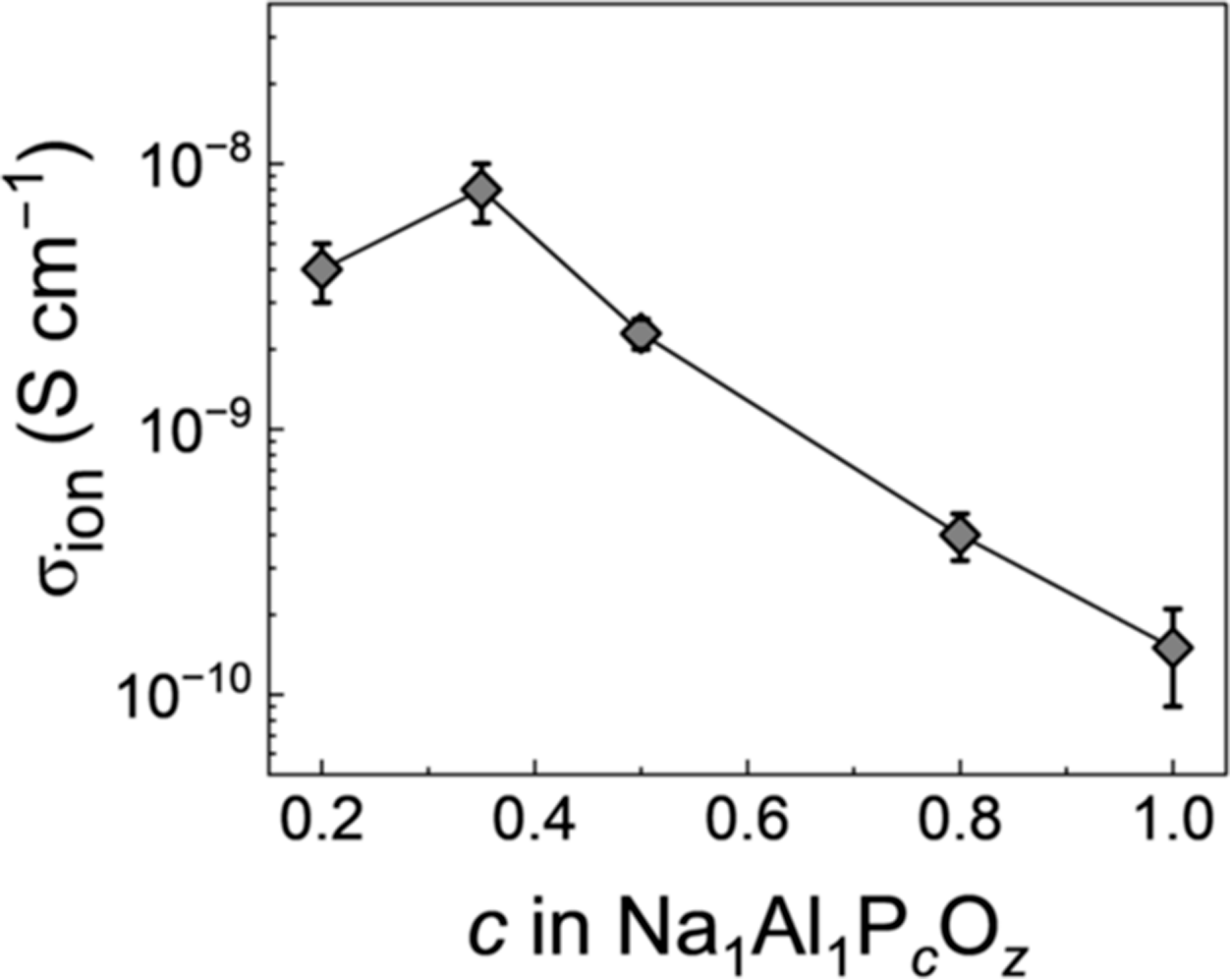
Room temperature σ_ion_ with error bars calculated using ±1 standard deviation. A final annealing temperature of 275 °C was used for all films.

To explore this further, we fixed the P content at *c* = 0.35 and 0.8 and then varied the Na content in the Na_*a*_Al_1_P_0.35_O_*z*_ and Na_*a*_Al_1_P_0.8_O_*z*_ films between 0.5 < *a* < 1.5 and 1.0 < *a* < 2.5 respectively. The GI-XRD data (Figure 6a) demonstrated an absence of the NaNO_3_ peaks at Na content, *a* = 0.5 and 0.75 (P = 0.35, red) and *a* = 1.0 and 1.25 (P = 0.8, blue), indicating complete integration of the Na^+^ ions from the NaNO_3_ precursor into amorphous Na_0.5–0.75_Al_1_P_0.35_O_*z*_ and Na_1.0–1.25_AlP_0.8_O_*z*_ matrices. Increasing the Na content to *a* = 1.0 (P = 0.35) and a = 1.5 (P = 0.8) results in the emergence of crystalline NaNO_3_ peaks, indicating matrix saturation. When the Na content is further increased to *a* = 1.25 and 1.5 (*c* = 0.35), and *a* = 2.0 and 2.5 (*c* = 0.8), increased XRD peak intensities, along with the emergence of additional peaks consistent with NaNO_3_ at 32° and 48° were observed. Comparison between the GI-XRD data sets suggests a clear link between the P content (*c*) in the precursor solution and the amount of Na (*a*) that can be integrated into the Al–P_*c*_–O_*z*_ matrices. Figure 6b displays the σ_ion_ determined from the fitted EIS data collected for all compositions. The σ_ion_ for Na_*a*_Al_1_P_0.35_O_*z*_ films increased from to 5(1) × 10^–10^ S cm^–1^ to 1.1(1) × 10^–8^ S cm^–1^ as the Na content was increased from *a* = 0.5 to 1.25, with a subsequent decrease to 2(1) × 10^–9^ S cm^–1^ at *a* = 1.5. Similarly, the σ_ion_ for Na_*a*_Al_1_P_0.8_O_*z*_ films increased from 4.0(8) × 10^–10^ S cm^–1^ to 1.0(2) × 10^–8^ S cm^–1^ as the Na content was increased from *a* = 1.0 to 2.0, with a reduction to 1.3(7) × 10^–9^ S cm^–1^ at *a* = 2.5. For both systems, an optimum σ_ion_ ≈ 10^–8^ S cm^–1^ was achieved by increasing the concentration of the NaNO_3_ content in the precursor solutions past the point of the matrix saturation, indicating that to some extent, the addition of an ionically insulating NaNO_3_ phase is beneficial to the σ_ion_.

**Figure 6:**
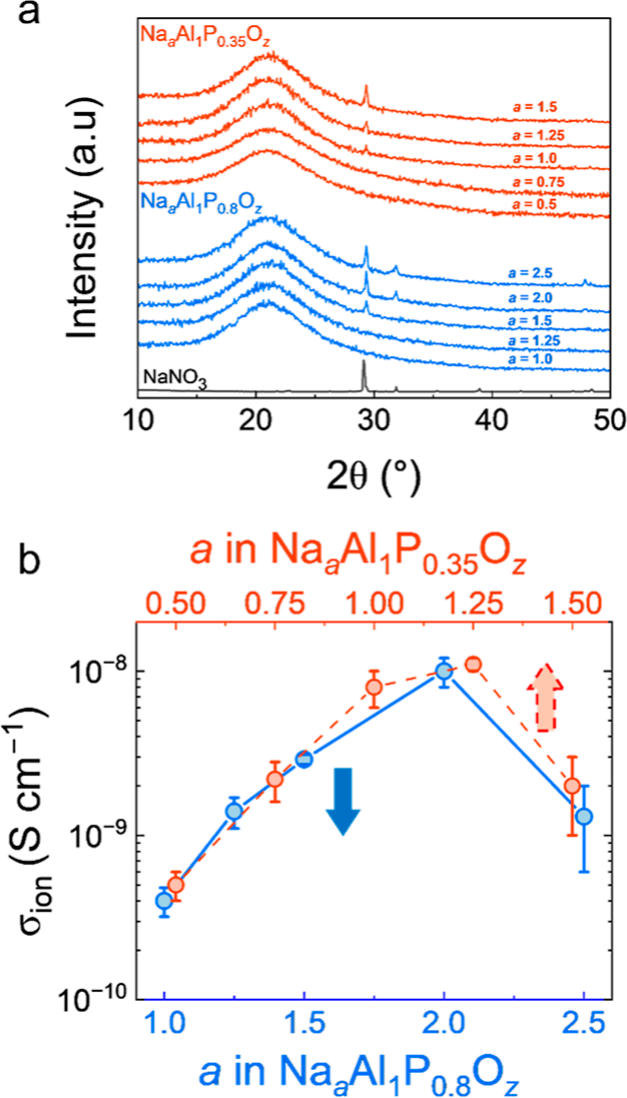
(a) GI-XRD data for Na_*a*_Al_1_P_*c*_O_*z*_ films with fixed P values (*c* = 0.35 and 0.8) and varied Na values. (b) Calculated room temperature σ_ion_ values for the various NAPO compositions, with error bars calculated using ±1 standard deviation. A final annealing temperature of 275 °C was used for all films.

### 3.4 Nanocomposite Structure

To gain information on the structure of the nanocomposites, STEM was conducted on powder scratched from a Na_2.5_Al_1_P_0.8_O_*z*_ film. The bright-field image (Figure 7) displays multiple, dark, highly diffracting (crystalline) nanoparticles (50–200 nm) embedded in a transparent (amorphous) matrix. Together, with the GI-XRD data this is consistent with crystalline NaNO_3_ nanoparticles dispersed within an amorphous NAPO matrix.

**Figure 7:**
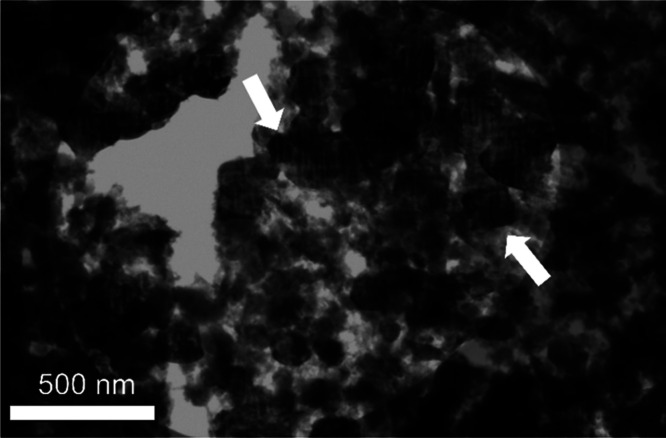
STEM bright-field image of Na_2.5_Al_1_P_0.8_O_*z*_. The white arrows indicate crystalline domains.

ToF-SIMS 3D analysis was then conducted on 4-layer films with the compositions Na_0.5_Al_1_P_0.35_O_*z*_, Na_1.0_Al_1_P_0.35_O_*z*_, and Na_1.5_Al_1_P_0.35_O_*z*_ (Figure 8). The collected secondary ions showed a uniform distribution of Na^+^, Al^+^ and PO_3_^–^ throughout all films, representing the homogeneous NAPO matrix. The depth profiles and 2D area maps for collected NO_3_^–^ ions for all 3 samples are displayed in Figure 8a,b, together with PO_3_^–^ ions for comparison. The presence of NaNO_3_ was indicated by the presence of NO_3_^–^ ions. It is noted that the entire film thickness was sputtered through after an ion dose of 3 × 10^15^ ions cm^–2^, but analyses was limited to 1.5 × 10^15^ ions cm^–2^ to allow for data collection in a reasonable time frame. For the Na_0.5_Al_1_P_0.35_O_*z*_ sample, there was some presence of NO_3_^–^ in the spectrum, however this was limited to the top surface. When increasing the Na content to Na_1.0_Al_1_P_0.35_O_*z*_, a higher intensity of NO_3_^–^ ions was observed at the top surface, which reduced quickly within the bulk of the films. The 2D area map displayed domains rich in NO_3_^–^ that were 10–50 μm in size. The primary ion beam, C_60_^+^ used for ToF-SIMS analysis was focused to ≈2 μm, which impacted the ability to distinguish particles smaller than this, however this is consistent with these areas being rich in NaNO_3_ nanoparticles. Upon increasing the Na content to Na_1.5_Al_1_P_0.35_O_*z*_, an increased presence of NO_3_^–^ was evident in the depth profiles, which showed a higher intensity of NO_3_^–^ ions that become negligible after about one-third of the film thickness. The 2D maps show ≈10 μm particles which are randomly distributed and separated across the material’s surface.

**Figure 8:**
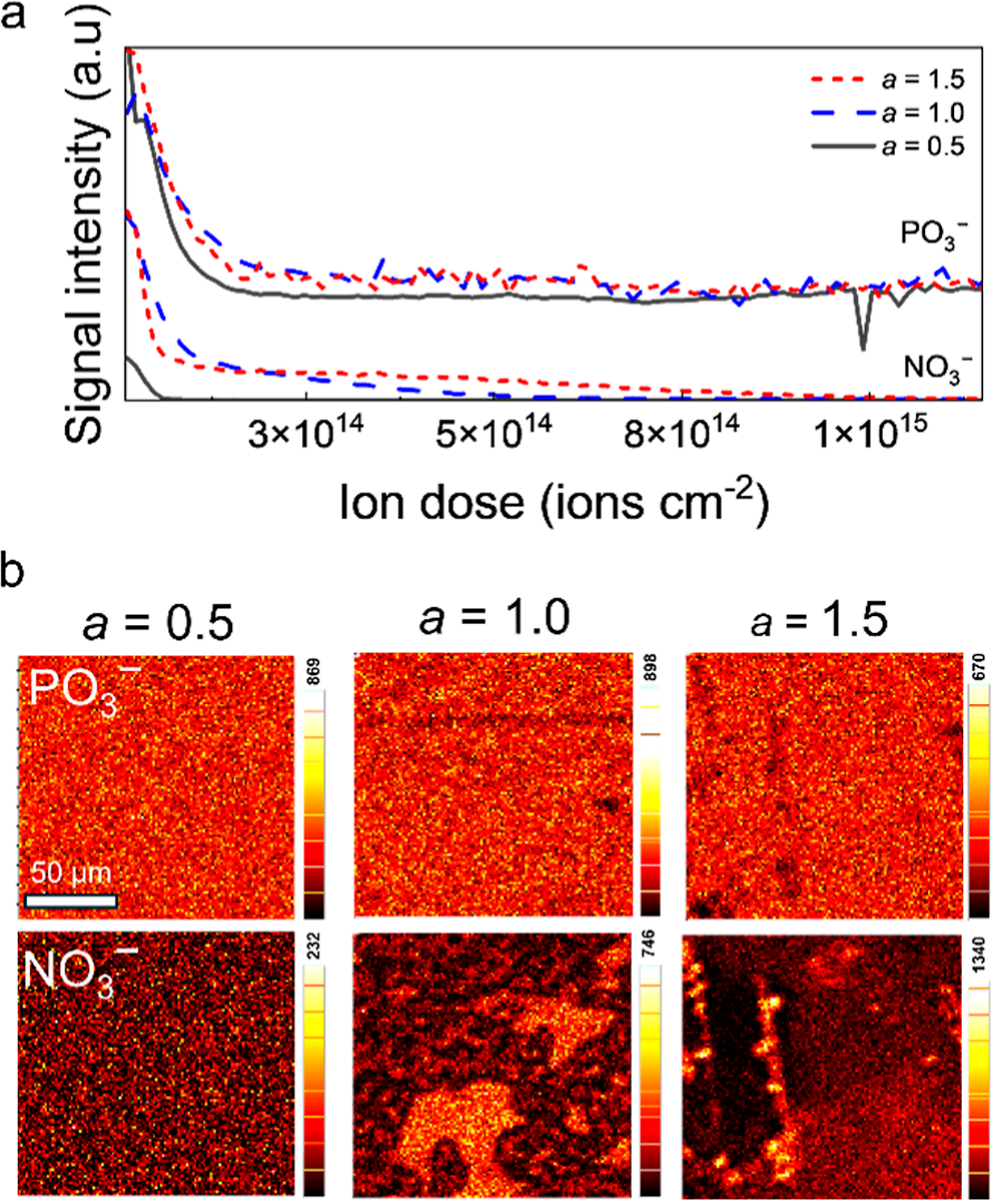
ToF-SIMS (a) depth profiles and (b) primary layer 2D area maps of secondary NO_3_^–^ and PO_3_^–^ ions Na_*a*_Al_1_P_0.35_O_*z*_ films with varied *a* content. An intensity scale with the maximum count level is shown for each 2D area map.

Taken altogether, the GI-XRD, STEM and ToF-SIMS data suggest that as the NaNO_3_ begins to crystallize out of the amorphous matrix, it starts as small particles which group together in relatively large domains in close proximity to the film surface. Upon further increase of the NaNO_3_ content, there is a preference for the crystals to group together in isolated clusters—likely made up of ≈100 nm size crystals as indicated by the STEM analysis. These clusters are embedded at the materials surface and extend further down into the bulk. These are displayed in the 3D volume maps generated from the ToF-SIMS data (Figure S5, Supporting Information).

The optical properties of the materials were also measured via diffuse reflectance analysis (Figure 9). The dip in reflectance near 400 nm is due to light absorption by NaNO_3_, which has been reported to have a band gap close to 3 eV.^39^ As the Na content in Na_*a*_Al_1_P_0.35_O_*z*_ films was increased from *a* = 0.5 to 1.25, the increasing surface coverage by uniformly distributed NaNO_3_ crystals results in greater surface roughness. This causes the observed increase in diffuse reflectance for these samples. When *a* = 1.5, the low-wavelength reflectance peak experiences a sudden drop in intensity. We propose that at the highest concentration of NaNO_3_, the aggregation of crystals into fewer isolated clusters smoothens the surface on average. This results in more specular behavior and a reduced diffuse scattering of incoming light. The increase in reflectance observed at higher wavelengths is due to the deeper extension of these clusters into the bulk of the material, where infrared radiation is more penetrative than visible and UV light.

**Figure 9:**
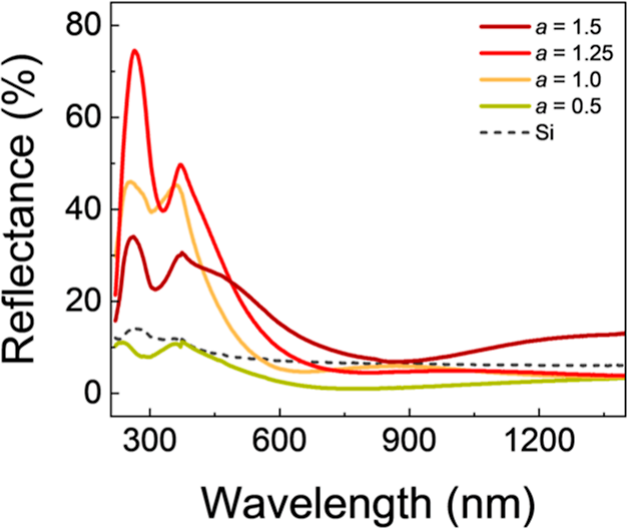
Reflectance data for Na_a_Al_1_P_0.35_O_*z*_ films with varied *a* content, together with Si substrate for reference.

Returning to the trends of σ_ion_, in the oversaturated two-phase Na_1.5_Al_1_P_0.35_O_*z*_ material, the extensive presence of the 10 μm NaNO_3_ particles negatively impacts the σ_ion_. On the other hand, in an intermediate-two phase system the NAPO matrix is saturated with Na^+^ ions, and the reduced concentration of the insulating NaNO_3_ phase (dispersed in subsurface domains) has a limited impact on σ_ion_. However, the addition of ionically insulating particles (e.g., SiO_2_ or Al_2_O_3_) has been reported to enhance the σ_ion_ when integrated into composites with numerous crystalline ion conductors.^40^ The exact reason for improved σ_ion_ is unknown but could be due to favorable changes in the crystal structure, lattice defects, and enhanced conduction pathways along a matrix–particle interface created by the dispersion of this second phase. The enhanced σ_ion_ observed in this work may therefore also be a result of these favorable pathways formed by the NaNO_3_ particles. However, it could also be due to formation of an ideal Na–Al–P–O composition that just happens to exsolve NaNO_3_. Future work will look to better understand the underlying mechanism.

For the materials with strong XRD peaks for NaNO_3_ (Na_1.25–1.5_Al_1_P_0.35_O_*z*_ and Na_2.0–2.5_Al_1_P_0.8_O_*z*_), visual color changes during prolonged exposure to ambient air indicated moisture contamination. The high content of NaNO_3_ in these materials will likely lead to increased moisture sensitivity, making them tougher to process and handle in ambient air. Therefore, the optimum composition was selected as Na_1_Al_1_P_0.35_O_*z*_, with an σ_ion_ = 8(2) × 10^–9^ S cm^–1^. Surface studies with AFM were reconducted (Figure S6, Supporting Information), demonstrating a low average roughness (<1 nm), and a reduced Young’s modulus of ≈30 GPa. For the subsequent charge transport investigations, this composition was chosen, and unless stated otherwise, the abbreviation NAPO will refer to the composition Na_1_Al_1_P_0.35_O_*z*_ annealed at 275 °C.

### 3.5 Activation Energy Analysis

The activation energy (*E*_a_) of the ion conduction process was calculated for optimized NAPO as 0.80(1) eV, using an Arrhenius relationship (Figure 10). This value is larger than values reported for LiPON (0.55 eV)^41^ and NaPON (0.53 eV),^18^ which is unsurprising given the comparatively lower σ_ion_ (NAPO ≈10^–8^ S cm^–1^, LiPON ≈10^–6^ S cm^–1^, NaPON ≈10^–7^ S cm^–1^). This value is greater than the value reported for a bulk sodium aluminophosphate glass (0.67 eV),^37^ however, a σ_ion_ value of ≈10^–8^ S cm^–1^ was achieved only at an elevated temperature (75 °C), at which NAPO demonstrated a superior σ_ion_ = 1.5 × 10^–7^ S cm^–1^ (70 °C). The comparatively higher ionic conductivity, despite the greater activation energy, could be explained by an increased concentration of mobile Na^+^ ions within the NAPO material.

**Figure 10:**
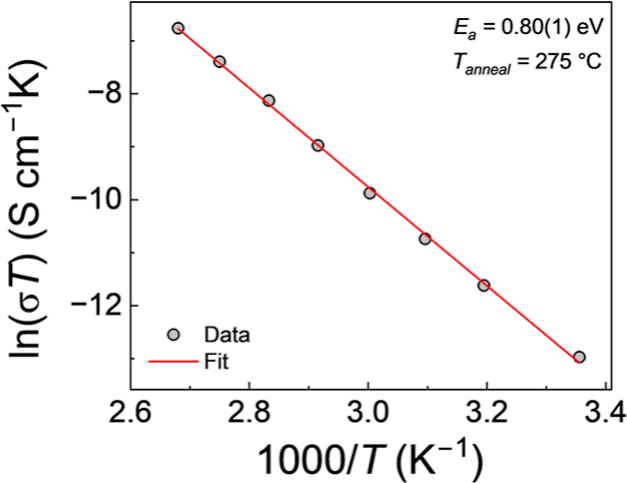
Temperature dependent σ_ion_ measurements from 25 to 100 °C. Calculated *E*_a_ inset, with error determined from the standard error as reported by the Origin fitting software.

### 3.6 Electronic Conductivity

Low bulk electronic conductivities have been shown to play an important role in resisting growth of alkali metal dendrites, and enabling stable plating and stripping of Li for example.^11^ From the steady-state current value of a voltage decay curve (Figure 11) the electronic conductivity of the NAPO film was calculated as ≈10^–14^ S cm^–1^, which compares well to the values reported for LiPON and water-processed LAPO films (≈10^–11^ to 10^–14^ S cm^–1^).^11,22^ Therefore, these materials are promising as artificial solid electrolyte interfaces or as SEs in alkali metal batteries.

**Figure 11:**
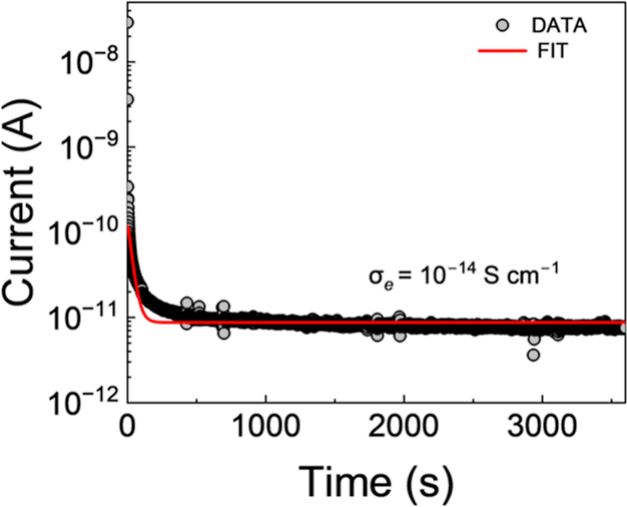
Current–voltage decay curve for the optimal NAPO film annealed at 275 °C.

## 4 Conclusions

Sodium thin film solid electrolytes were synthesized via spin coating from aqueous precursors. First, the physical, structural, and electrochemical properties were benchmarked using a Na_1_Al_1_P_0.5_O_*z*_ composition. A 275 °C anneal was sufficient to form continuous, dense thin films with low surface roughness (<1 nm) and a moderate Young’s modulus. XPS and XRD analyses indicated the presence of residual NaNO_3_ precursor salt which was decomposed after an 1 h anneal at 500 °C. The Na–Al–P–O phase space was then explored, which revealed the existence of two-phase compositions, made up of an amorphous NAPO phase and crystalline NaNO_3_ precursor domains. A maximum σ_ion_ of ≈10^–8^ S cm^–1^ was reported in three of these nanocomposites: Na_1_Al_1_P_0.35_O_*z*_, Na_1.25_Al_1_P_0.35_O_*z*_ and Na_2.0_Al_1_P_0.8_O_*z*_. Although the role of the insulating NaNO_3_ crystallites on the σ_ion_ is not fully understood, it is clear that partially increasing their presence within the films is desirable. Temperature dependent σ_ion_ measurements for the Na_1_Al_1_P_0.35_O_*z*_ materials exhibited an activation energy of 0.80(1) eV and a low σ_e_ (≈10^–14^ S cm^–1^). These results should aid in designing new amorphous and amorphous-ceramic solid electrolytes for alkali metal batteries.

## Supplementary Material



## Notes

Surface elemental ratios determined via XPS, ellipsometry data, EIS data, 3D ToF-SIMS data, and AFM surface profile (PDF)

## References

[1] Lee B., Paek E., Mitlin D., Lee S. W. (2019). Sodium Metal Anodes:
Emerging Solutions to Dendrite Growth. Chem.
Rev..

[2] Huang J., Wu K., Xu G., Wu M., Dou S., Wu C. (2023). Recent Progress
and Strategic Perspectives of Inorganic Solid Electrolytes: Fundamentals,
Modifications, and Applications in Sodium Metal Batteries. Chem. Soc. Rev..

[3] Liu G., Yang J., Wu J., Peng Z., Yao X. (2024). Inorganic
Sodium Solid Electrolytes: Structure Design, Interface Engineering
and Application. Adv. Mater..

[4] Tsai C. L., Lan T., Dellen C., Ling Y., Ma Q., Fattakhova-Rohlfing D., Guillon O., Tietz F. (2020). Dendrite-Tolerant All-Solid-State
Sodium Batteries and an Important Mechanism of Metal Self-Diffusion. J. Power Sources.

[5] Fu H., Yin Q., Huang Y., Sun H., Chen Y., Zhang R., Yu Q., Gu L., Duan J., Luo W. (2020). Reducing Interfacial
Resistance by Na-SiO2 Composite Anode for NASICON-Based Solid-State
Sodium Battery. ACS Mater. Lett..

[6] Zhou W., Li Y., Xin S., Goodenough J. B. (2017). Rechargeable Sodium All-Solid-State
Battery. ACS Cent. Sci..

[7] Miao X., Di H., Ge X., Zhao D., Wang P., Wang R., Wang C., Yin L. (2020). AlF3-Modified Anode-Electrolyte Interface
for Effective Na Dendrites Restriction in NASICON-Based Solid-State
Electrolyte. Energy Storage Mater..

[8] Bay M. C., Wang M., Grissa R., Heinz M. V. F., Sakamoto J., Battaglia C. (2020). Sodium Plating
from Na-β″-Alumina Ceramics
at Room Temperature, Paving the Way for Fast-Charging All-Solid-State
Batteries. Adv. Energy Mater..

[9] Li J., Ma C., Chi M., Liang C., Dudney N. J. (2015). Solid Electrolyte:
The Key for High-Voltage Lithium Batteries. Adv. Energy Mater..

[10] Bates J. B., Dudney N. J., Neudecker B., Ueda A., Evans C. D., ThinBates J. B., Dudney N. J., Neudecker B., Ueda A., Evans C. D. (2000). Thin-Film Lithium and Lithium-Ion
Batteries. Solid State Ionics.

[11] Han F., Westover A. S., Yue J., Fan X., Wang F., Chi M., Leonard D. N., Dudney N. J., Wang H., Wang C. (2019). High Electronic
Conductivity as the Origin of Lithium Dendrite Formation within Solid
Electrolytes. Nat. Energy.

[12] Krauskopf T., Richter F. H., Zeier W. G., Janek J. (2020). Physicochemical Concepts
of the Lithium Metal Anode in Solid-State Batteries. Chem. Rev..

[13] Cheng D., Wynn T. A., Wang X., Wang S., Zhang M., Shimizu R., Bai S., Nguyen H., Fang C., KimLi M. W., Lu B., Kim S. J., Meng Y. S., Meng Y. S. (2020). Unveiling the Stable Nature of the
Solid Electrolyte
Interphase between Lithium Metal and LiPON via Cryogenic Electron
Microscopy. Joule.

[14] Su J., Pasta M., Ning Z., Gao X., Bruce P. G., Grovenor C. R. M. (2022). Interfacial Modification between
Argyrodite-Type Solid-State
Electrolytes and Li Metal Anodes Using LiPON Interlayers. Energy Environ. Sci..

[15] Lee S., Jung S., Yang S., Lee J. H., Shin H., Kim J., Park S. (2022). Revisiting the LiPON/Li Thin Film as a Bifunctional
Interlayer for NASICON Solid Electrolyte-Based Lithium Metal Batteries. Appl. Surf. Sci..

[16] Liu W., Guo R., Zhan B., Shi B., Li Y., Pei H., Wang Y., Shi W., Fu Z., Xie J. (2018). Artificial
Solid Electrolyte Interphase Layer for Lithium Metal Anode in High-Energy
Lithium Secondary Pouch Cells. ACS Appl. Energy
Mater..

[17] Wang W., Yue X., Meng J., Wang J., Wang X., Chen H., Shi D., Fu J., Zhou Y., Chen J., Fu Z. (2019). Lithium Phosphorus
Oxynitride as an Efficient Protective Layer on Lithium Metal Anodes
for Advanced Lithium-Sulfur Batteries. Energy
Storage Mater..

[18] Nuwayhid R. B., Jarry A., Rubloff G. W., Gregorczyk K. E. (2020). Atomic
Layer Deposition of Sodium Phosphorus Oxynitride: A Conformal Solid-State
Sodium-Ion Conductor. ACS Appl. Mater. Interfaces.

[19] Nuwayhid R. B., Fontecha D., Kozen A. C., Jarry A., Lee S. B., Rubloff G. W., Gregorczyk K. E. (2022). Nanoscale
Li, Na, and K Ion-Conducting
Polyphosphazenes by Atomic Layer Deposition. Dalton Trans..

[20] Nuwayhid R. B., Kozen A. C., Long D. M., Ahuja K., Rubloff G. W., Gregorczyk K. E. (2023). Dynamic Electrode-Electrolyte Intermixing in Solid-State
Sodium Nano-Batteries. ACS Appl. Mater. Interfaces.

[21] Clayton D. R., Lepage D., Plassmeyer P. N., Page C. J., Lonergan M. C. (2017). Low-Temperature
Fabrication of Lithium Aluminum Oxide Phosphate Solid Electrolyte
Thin Films from Aqueous Precursors. RSC Adv..

[22] Vadhva P., Gill T. E., Cruddos J. H., Said S., Siniscalchi M., Narayanan S., Pasta M., Miller T. S., Rettie A. J. E. (2023). Engineering
Solution-Processed Non-Crystalline Solid Electrolytes for Li Metal
Batteries. Chem. Mater..

[23] Zhang Z., Smith K., Jervis R., Shearing P. R., Miller T. S., Brett D. J. L. (2020). Operando Electrochemical
Atomic Force Microscopy of
Solid-Electrolyte Interphase Formation on Graphite Anodes: The Evolution
of SEI Morphology and Mechanical Properties. ACS Appl. Mater. Interfaces.

[24] Zhang Z., Said S., Lovett A. J., Jervis R., Shearing P. R., Brett D. J. L., Miller T. S. (2024). The Influence of Cathode Degradation
Products on the Anode Interface in Lithium-Ion Batteries. ACS Nano.

[25] Said S., Zhang Z., Shutt R. R. C., Lancaster H. J., Brett D. J. L., Howard C. A., Miller T. S. (2023). Black Phosphorus
Degradation during Intercalation and Alloying in Batteries. ACS Nano.

[26] Nečas D., Klapetek P. (2012). Gwyddion: An Open-Source Software for SPM Data Analysis. Cent. Eur. J. Phys..

[27] Fletcher J. S., Rabbani S., Henderson A., Blenkinsopp P., Thompson S. P., Lockyer N. P., Vickerman J. C. (2008). A New Dynamic
in Mass Spectral Imaging of Single Biological Cells. Anal. Chem..

[28] Randau S., Weber D. A., Kötz O., Koerver R., Braun P., Weber A., Ivers-Tiffée E., Adermann T., Kulisch J., Zeier W. G., Richter F. H., Janek J. (2020). Benchmarking the Performance
of All-Solid-State Lithium Batteries. Nat. Energy.

[29] Lasia, A. Electrochemical Impedance Spectroscopy and Its Applications; Springer New York, NY, 2014; pp 1–367.

[30] Westerhoff U., Kurbach K., Lienesch F., Kurrat M. (2016). Analysis of Lithium-Ion
Battery Models Based on Electrochemical Impedance Spectroscopy. Energy Technol..

[31] Meyers S. T., Anderson J. T., Hong D., Hung C. M., Wager J. F., Keszler D. A. (2007). Solution-Processed Aluminum Oxide Phosphate Thin-Film
Dielectrics. Chem. Mater..

[32] Yuvaraj S., Fan-Yuan L., Tsong-Huei C., Chuin-Tih Y. (2003). Thermal Decomposition
of Metal Nitrates in Air and Hydrogen Environments. J. Phys. Chem. B.

[33] Herbert E. G., Tenhaeff W. E., Dudney N. J., Pharr G. M. (2011). Mechanical Characterization
of LiPON Films Using Nanoindentation. Thin Solid
Films.

[34] Yu S., Schmidt R. D., Garcia-Mendez R., Herbert E., Dudney N. J., Wolfenstine J. B., Sakamoto J., Siegel D. J. (2016). Elastic Properties
of the Solid Electrolyte Li7La3Zr2O12 (LLZO). Chem. Mater..

[35] Kato A., Nagao M., Sakuda A., Hayashi A., Tatsumisago M. (2014). Evaluation
of Young’s Modulus of Li2S-P2S 5-P2O5 Oxysulfide Glass Solid
Electrolytes. J. Ceram. Soc. Jpn..

[36] Hayashi A., Sakuda A., Tatsumisago M. (2016). Development
of Sulfide Solid Electrolytes
and Interface Formation Processes for Bulk-Type All-Solid-State Li
and Na Batteries. Front. Energy Res..

[37] Keshri S. R., Ganisetti S., Kumar R., Gaddam A., Illath K., Ajithkumar T. G., Balaji S., Annapurna K., Nasani N., Krishnan N. M. A., Allu A. R. (2021). Ionic Conductivity
of Na3Al2P3O12Glass Electrolytes - Role of Charge Compensators. Inorg. Chem..

[38] Barik S. K., Senapati A., Chakraborty S., Ananthasivan K. (2023). Structure
and Optical Properties of Sodium Aluminium Phosphate Glass Matrix
Containing Lanthanide Oxides (Ce, Pr, Nd and Gd). J. Inorg. Organomet. Polym. Mater..

[39] Balabinskaya A. S., Ivanova E. N., Ivanova M. S., Kumzerov Y. A., Pan’Kova S. V., Poborchii V. V., Romanov S. G., Solovyev V. G., Khanin S. D. (2005). Investigation
into the Electrical and Optical Properties of Sodium Nitrite and Sodium
Nitrate Nanoparticles in Regular Porous Matrices. Glass Phys. Chem..

[40] Dudney N. J. (1989). Composite
Electrolytes. Annu. Rev. Mater. Sci..

[41] Lacivita V., Artrith N., Ceder G. (2018). Structural
and Compositional Factors
That Control the Li-Ion Conductivity in LiPON Electrolytes. Chem. Mater..

