# Supplementary material for: Saturating the Matrix: Nanocomposite Solution-Processed Sodium Aluminophosphate Solid Electrolytes
Source: ACS Appl Energy Mater. 2025 Oct 1;8(19):14592–600. doi: 10.1021/acsaem.5c02284 (PMC12522096; doi:10.1021/acsaem.5c02284)
Supplement: Supplementary file 1 [file ae5c02284_si_001.pdf]

Supporting Information for:

Saturating the Matrix: Nanocomposite Solution-Processed Sodium Aluminophosphate Solid Electrolytes

Thomas E. Gill,[a,b] Guillaume Matthews,[c] Yaoguang Song,[a,b] Mo El Maoued,[d] Adam J. Lovett,[a,b] Sadia Sheraz,[e] Nicholas P. Lockyer,[e] Amita Ummadisingu,[d] Thomas S. Miller,[a,b] Alexander J. E. Rettie[a,b,*]

[a] Electrochemical Innovation Lab, Department of Chemical Engineering, University College London, WC1E 6DH (UK)

[b] Advanced Propulsion Lab, University College London, Marshgate, Stratford, E20 2AE (UK)

[c] Department of Materials, University of Oxford, Oxford, OX1 3PH (UK)

[d] Manufacturing Futures Lab, Department of Chemical Engineering, University College London, WC1E 6DH (UK)

[e] Department of Chemistry, Photon Science Institute, The University of Manchester, Oxford Road, Manchester, M13 9PL, U.K.

* For correspondence: a.rettie@ucl.ac.uk

Table S1: Elemental ratios determined from fitted peaks in XPS

Precursor Ratio (Na:Al:P)	Annealing T (C)	Na 1s	Al 2p	P 2p	O 1s	N 1s
1:1:0.5	275	0.77(10)	1	0.51(2)	2.6(2)	0.07(2)
1:1:0.5	500	0.77(4)	1	0.55(2)	2.8(2)	0

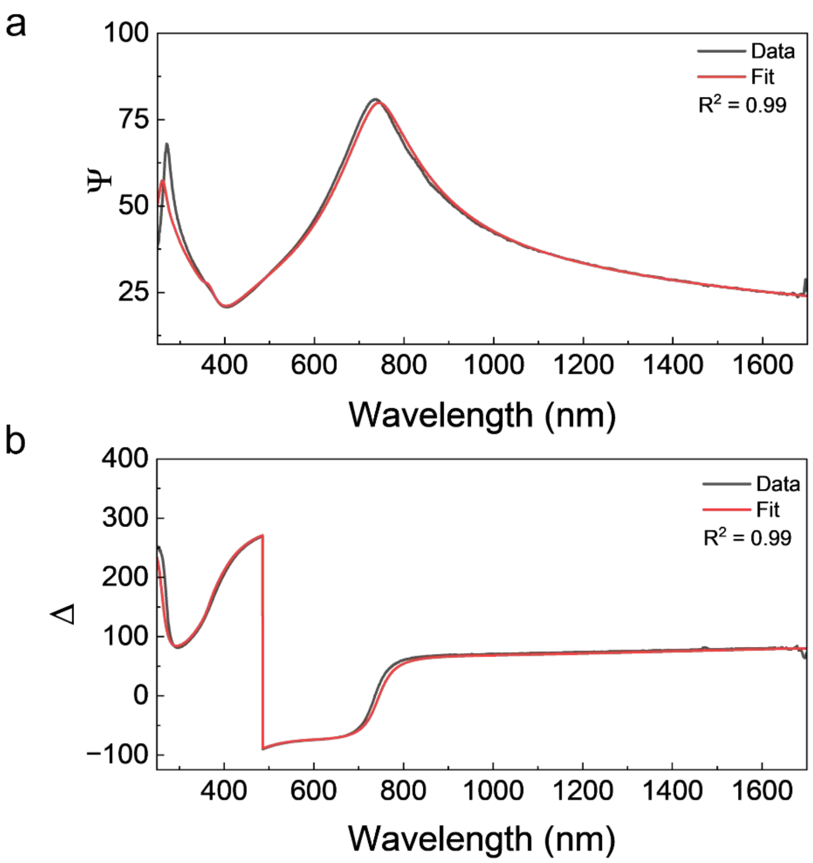

Figure S1: Measured and fitted ellipsometry data for (a) Ψ and (b) Δ parameters for a 2 layer NAPO film.

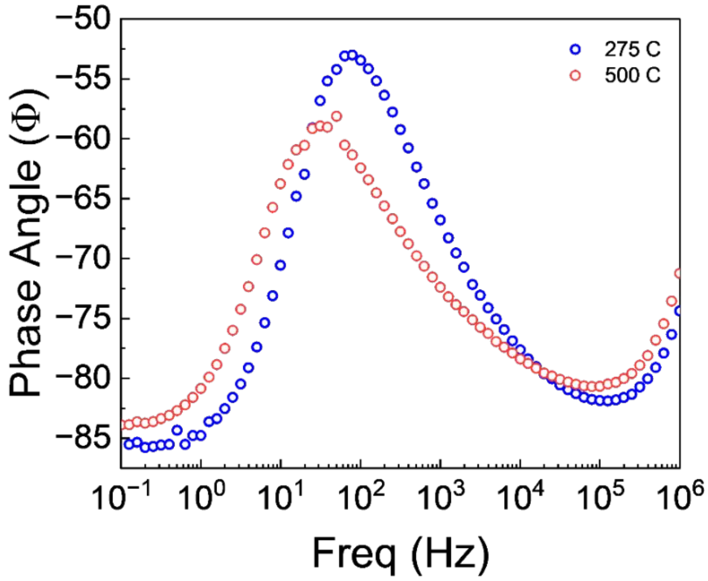

Figure S2: BODE plot for $Na_1Al_1P_{0.5}O_z$ films annealed at 275 ℃ and 500 ℃

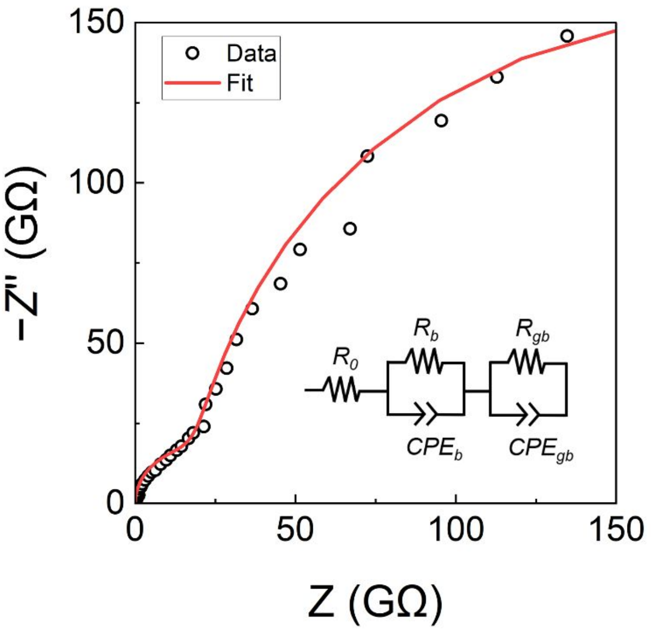

Figure S3: EIS Data for a NaNO3 pellet with ECM fit inset, recorded at 40 ℃.

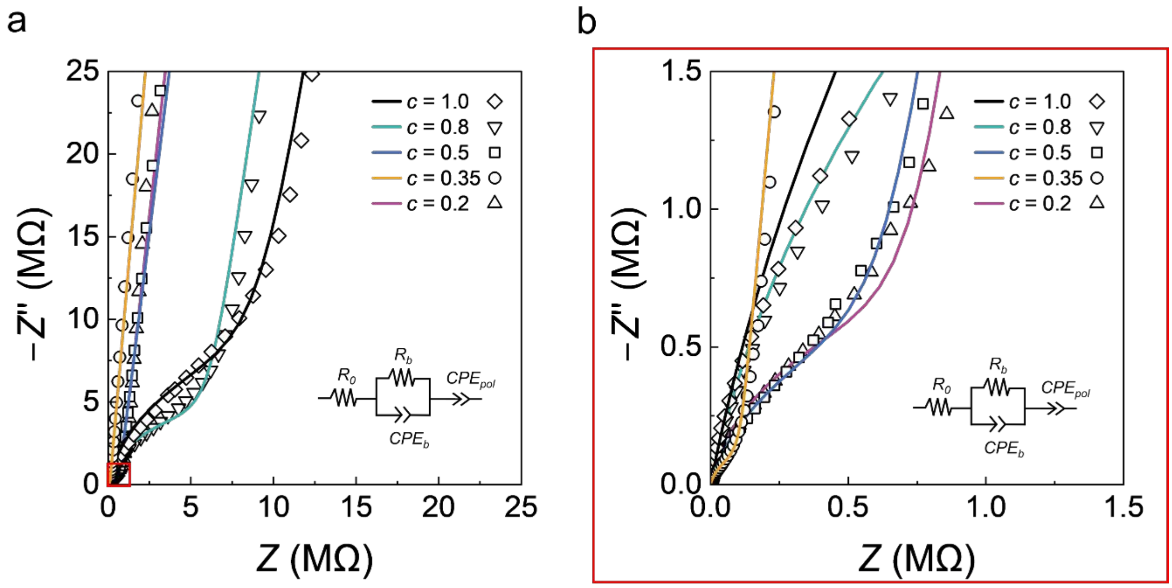

Figure S4: (a) EIS data for $Na_1Al_1P_cO_z$ films with varied P content from $0.2 < c < 1.0$ and a fixed annealing temperature (275 ℃) with equivalent circuit model used to fit the data.(b) A close up of the same data focused on the lower resistance range (red square in (a))

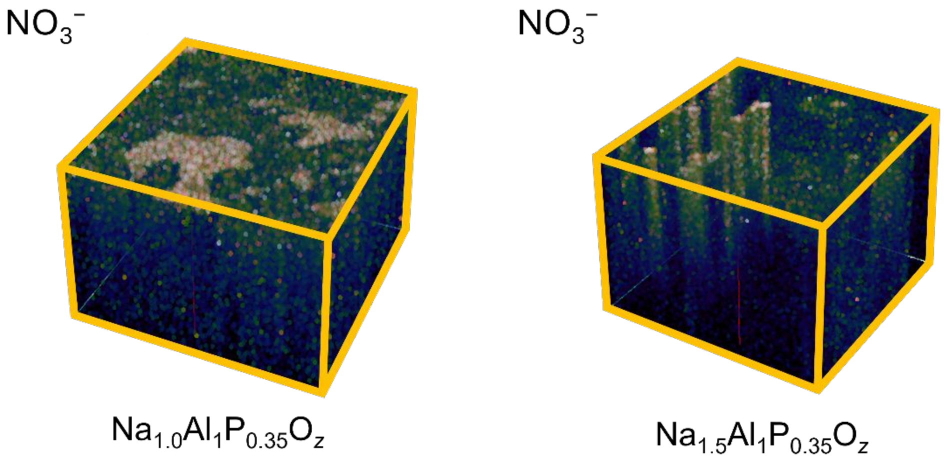

Figure S5: 3D volume maps on the collected NO_3^- ion fragments for $Na_1Al_1P_{0.35}O_z$ and $Na_{1.5}Al_1P_{0.35}O_z$ samples. Note here the xy plane is 150 μm × 150 μm, and ≈150 nm of material was sputtered after total dose of 1.5×10^{15} ions cm^{-2}, therefore the z axes has been stretched for visualisation.

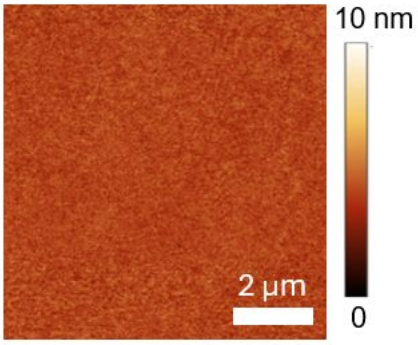

Figure S6: AFM surface profile for optimal $Na_1Al_1P_{0.35}O_z$ film.
